# Health Policy and Screening for Colorectal Cancer in the United States

**DOI:** 10.3390/cancers17122003

**Published:** 2025-06-16

**Authors:** Maryam R. Hussain, Faisal S. Ali, Scott A. Larson, Soham Al Snih

**Affiliations:** 1 Department of Population Health and Health Disparities, School of Public and Population Health, University of Texas Medical Branch, Galveston, TX 77555, USA; mrhussai@utmb.edu; 2 Department of Gastroenterology, Beth Israel Deaconess Medical Center, Harvard Medical School, Boston, MA 02115, USA; 3 Section of Gastroenterology and Hepatology, Department of Medicine, Kelsey-Seybold Clinic, Houston, TX 77025, USA; 4 Division of Geriatrics, Department of Internal Medicine, University of Texas Medical Branch, Galveston, TX 77555, USA

**Keywords:** colorectal cancer screening, colonoscopy, FIT test, ACA, Medicare, Medicaid

## Abstract

The landscape for the screening of colorectal cancer (CRC) has witnessed multiple triumphs over the past decades from policy-level interventions. In the United States (US), the most prominent intervention of this nature is the Patient Protection and Affordable Care Act (ACA), enacted more than a decade ago. Since its enactment, the ACA has seen multiple legal challenges, and its impact on CRC screening has been relatively well studied. However, a consolidated, concise analysis of the data on this subject is lacking. Herein, we evaluate the impact of the ACA on CRC screening through the lens of a policy analysis, highlighting its strengths and shortcomings, and suggest policy-level interventions to address these shortcomings and improve CRC screening adoption.

## Simple Summary

Colorectal cancer (CRC) screening promotion benefits from support at a policy level, as is evident from analyses of data generated after the enactment of the Affordable Care Act (ACA). Improving national CRC rates requires a thorough understanding of loopholes within the ACA and the policies that followed; this is critical for the identification of obstacles that can be best addressed by additional target policy efforts. The current body of evidence hints at underutilization of the breadth of CRC screening modalities available for the public that are currently recommended by the United States Preventive Services Task Force (USPSTF). Particularly, underutilization of stool-based testing that can be mailed and performed at home may represent a missed opportunity that can be key to improving national screening rates. The promotion of a two-step screening strategy has historically been limited; cost-sharing for diagnostic colonoscopies after a positive non-invasive test may have been a considerable contributing factor. With recent policy efforts to address cost-sharing, the promotion of a two-step screening strategy may be central to improving CRC screening to reach the target rates put forth by the National Colorectal Cancer Roundtable consensus. There is a need to establish new HEDIS quality metrics, including the proportion of patients with an abnormal non-invasive CRC screening test with a follow-up colonoscopy (target completion rate of 80%), and the time to the completion of such a colonoscopy (target time to completion within 9 months).

## 1. Introduction

Colorectal cancer (CRC) is the third most diagnosed malignancy and is the second leading cause of cancer-related mortality in the United States [1]. CRC is also among the few cancers with a robust profile of screening modalities endorsed and recommended by the United States Preventive Services Task Force (USPSTF; Table 1) [2].

Cumulatively, CRC screening of any type may provide a 30–60% reduction in CRC incidence and mortality. Except for a colonoscopy, all CRC screening modalities involve a two-step process:

- Administration of an index screening test.
- Follow-up colonoscopy for a positive index test.

The Patient Protection and Affordable Care Act (ACA) was signed into law in March 2010 [2] and carried specific provisions under which CRC screening tests recommended by the USPSTF with a grade A or B strength should be provided to the public without cost-sharing or a deductible. Since its enactment, more than 2000 legal challenges contesting part or all of the ACA have been filed in state and federal courts [3]. Most recently, litigation in the state of Texas (Braidwood Management v. Becerra) asserted that the enforcement of recommendations put forth by specific expert committees such as the USPSTF and adopted broadly by the Federal Government to cover preventive services is unconstitutional, requesting a universal remedy to block the enforcement of such recommendations; these include the delivery of preventive health services, including CRC screening [4].

This review article addresses a critical need; to highlight the benefits and address the shortcomings of the ACA in the form of a policy analysis and to guide future policymaking to effectively reduce CRC-related morbidity and mortality.

## 2. Eligibility and Affordability of Care and the ACA

Under the ACA, individuals at all income levels can sign up for health insurance. A premium tax credit or subsidy is available for households with an income between 100 and 400% of the Federal Poverty Level (FPL). By 2022 FPLs, a family of four would qualify for subsidies with a household income of USD 27,750–111,000, whereas a single person would qualify for subsidies if they earned USD 13,590–54,360 [5]. As of 2022, the rate of uninsured Americans was at eight percent, historically the lowest. As of April 2022, over 35 million Americans had coverage through the ACA, and as of June 2022, 89.5 million Americans were enrolled in Medicaid and the Children’s Health Insurance Program (CHIP) [6]. All average-risk individuals who meet the criteria for CRC screening and are enrolled in these programs are eligible to receive screening, free of charge to the individual.

An analysis of the data from 2011 to 2016 of the National Health Interview Survey (NHIS) has shown that the implementation of the ACA’s insurance expansion provisions improved the financial well-being of individuals by decreasing healthcare-related financial strain [7]. The largest impact was observed among households earning between 0 and 124% and 125 and 199% of the FPL; this represents individuals who traditionally did not meet income eligibility requirements for public insurance programs. A reduction in the incidence of new medical debt, balances past due, the probability of medical bills going to debt collection, a medical collection balance of USD 1000 or more, the probability of a new bankruptcy filing, and a reduction in the utilization of payday loans—a high interest rate loan—along with improved credit scores have been reported in studies of Medicaid expansion states [8,9,10]. These data suggest an improvement in the financial well-being of individuals after the implementation of the ACA, either by direct or indirect means.

Financial toxicity is a term that reflects the economic burden of cancer care and takes into account material costs, the psychological impact of such costs, and the coping strategies adopted by individuals to curb such economic distress, which includes delaying or foregoing care [11]. Material costs are known to be driven by high out-of-pocket costs [12]. Data from the NHIS (9277 individuals ≥ 50 years of age; 5115 who had a screening colonoscopy and 4162 who did not) has emerged suggesting that higher financial hardship or toxicity, as measured by a financial hardship score derived from 10 questions pertaining to financial toxicity, is associated with a decreased probability of CRC screening [13]. This highlights the value of financial well-being and underscores the need for further study of the impact of economic well-being on the completion of CRC screening.

## 3. Policies Addressing Screening Before the ACA

**Figure 1:**
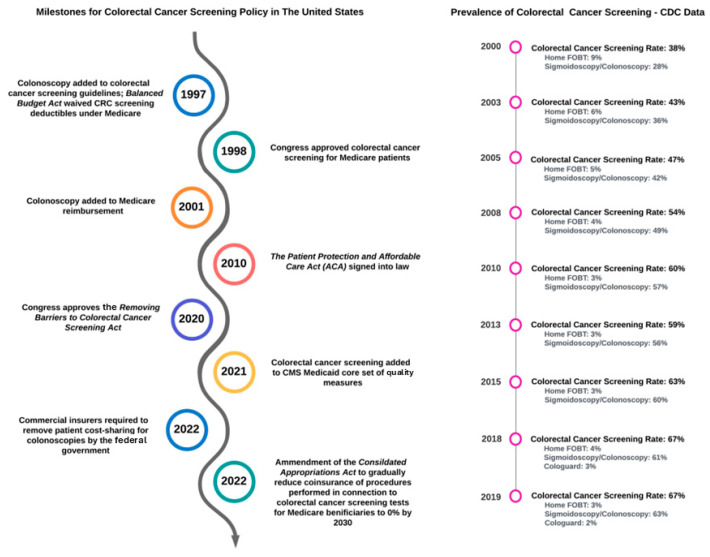
Chronological evolution of CRC screening policy and screening rates.

In the pre-ACA era (before 2010), over 50 million (one in six) Americans had no access to health insurance [14,15]. Medicare waived deductibles for CRC screening before the enactment of the ACA due to policy-level interventions such as the Balanced Budget Act of 1997 (Figure 1) [16] and the Consolidated Appropriations Act of 2001 [17], both of which aimed to reduce the national CRC burden by increasing coverage of screening modalities. These policies likely impacted CRC screening rates, which increased from 38% in 2000 to 60% in 2010. However, policies before the ACA did not explicitly address cost-sharing for screening tests.

## 4. Effectiveness of the ACA in Improving CRC Screening Rates

Studies on CRC screening after the enactment of the ACA are suggestive of an increase in the uptake of screening among beneficiaries of private insurance, Medicare, and Medicaid. It should, however, be noted that we are still in the honeymoon phase of the ACA in terms of population impact and cancer prevention; the data available to us at this point in time should therefore be interpreted within this context.

### 4.1. CRC Screening Uptake and Medicare

A study of the Medical Expenditure Panel Survey (MEPS) showed that compared to 2009, there was a 9 and 8% increase in CRC screening among Medicare beneficiaries in 2011 and 2014 (adjusted prevalence ratio (APR) in 2011: 1.09; 95% CI 1.02–1.16; APR in 2014: 1.08; 95% CI 1.01–1.15) [18]. Upon further stratification, the increase in prevalence was most prominent among males, with a 13% increase in CRC screening among men on Medicare in 2011 (APR: 1.13; 95% CI 1.02–1.25), which decreased to 9% in 2014 (APR: 1.09; 95% CI 0.98–1.21); no statistically significant increase in CRC screening was noted among women. Notably, Hispanic Medicare beneficiaries had a 40–44% increase in the uptake of CRC screening in 2011–2014, whereas non-Hispanic whites, non-Hispanic Blacks, and non-Hispanic Asians did not demonstrate a statistically significant increase in CRC screening [18]. Data from 5853 and 9933 CRC screening eligible adults in the pre-ACA (2008) and post-ACA (2013) period from the NHIS showed a 3.9% overall increase in CRC screening uptake between 2008 and 2013; the percent change was most prominent among Medicare beneficiaries, with a 9.8% increase from 2008 (*n* = 1251; screening rate: 50.45; 95% CI 47.1–53.6%) to 2013 (*n* = 2617; screening rate: 60.2%; 95% CI 57.7–62.7%) [19]. Of note, Medicare plans are required to report CRC screening rates as a measurable metric.

### 4.2. CRC Screening Uptake in Medicaid Expansion States

Data from Community Health Centers (CHC), a health system that serves a socioeconomically disadvantaged population, showed that among states that adopted Medicaid expansion, the prevalence of CRC screening increased from 11% pre-ACA (*n* = 92,821) to 18% post-ACA (*n* = 99,410); states without Medicaid expansion noted an increase in the prevalence of CRC screening from 13% pre-ACA (*n* = 77,135) to 21% post-ACA (*n* = 85,488), with no significant difference between states with and without Medicaid expansion [20]. Davis et al. specifically looked at the implementation of Accountable Care Organizations and Medicaid expansion within the State of Oregon; compared to pre-ACA rates (2010; number of eligible individuals: 20,333), a 3.2% increase in the prevalence of CRC screening was noted in 2014 (13% vs. 16.2%; number of eligible individuals: 114,681) [21]. Similarly, the analysis of data from the state of Kentucky showed that compared to pre-ACA rates (2011–2013; patients with Medicaid: 20,980), there was a 230% increase in the prevalence of CRC screening among Medicaid beneficiaries post-ACA (2013–2016; patients with Medicaid: 69,328) [22]. Analyses of the Behavioral Risk Factor Surveillance System (BRFSS) showed a statistically significant increase in CRC screening in states with Medicaid expansion prior to 2014 compared to non-expansion states [23,24,25]. An updated analysis of data from the BRFSS (2012–2020) showed that compared to 2012, in 2016, a crude increase of 16.45% in CRC screening was noted among respondents from very-early Medicaid expansion states [26]. However, from 2012 to 2020, CRC screening increased in all states regardless of Medicaid expansion status. Medicaid plans in expansion states, until recently, were not required to report CRC screening rates as a measurable metric [27]. Of note, the uptake of CRC screening, or lack thereof, in Medicaid populations is likely multifactorial and cannot be attributed solely to the lack of reporting of a quality metric.

### 4.3. CRC Screening Uptake and Commercial Insurance

Data from the Medical Expenditure Panel Survey (MEPS) showed that compared to 2009, a 6% increase in the prevalence of CRC screening was noted among beneficiaries of private insurance in 2011 (APR: 1.06; 95% CI 1.01–1.10) [18]. Per the ACA, private insurance plans starting on 23 September 2010 and Medicare plans starting 1 January 2011 were required to eliminate cost-sharing for screening modalities, including CRC screening; commercial plans that existed in March 2010 were “grandfathered” under the ACA and did not make any coverage changes. Data from the Humana beneficiaries’ claims database showed a 3.1% increase in screening colonoscopy rates from 2008 to 2012 among individuals with non-grandfathered plans (*n* = 25,926) compared to grandfathered plans (*n* = 37,320); this difference was not statistically significant [28]. An additional analysis of colonoscopies coded only as preventative also failed to show a statistically significant difference in the uptake of CRC screening over time between the two plan structures. Among the beneficiaries of United Healthcare with a high-deductible health plan, a 9.1% increase in the CRC screening rate was noted from 2003 to 2012 (absolute change per 10,000: 89; 95% CI 11–168) [29].

### 4.4. CRC Screening and Socioeconomic Status

Data obtained from the MEPS, the NHIS, and the BRFSS have demonstrated a statistically significant increase in CRC screening among low-income individuals, those living in poverty, and Medicaid-insured individuals, all populations deemed to be socioeconomically disadvantaged [19,30,31]. An analysis of the NHIS data showed that when stratified by income, an increase in CRC screening prevalence was most pronounced among individuals with an annual income of <USD 35,000 (4.3% difference between 2008 (screening prevalence 51%; 05% CI 48.1–53.8%) and 2013 (screening prevalence: 55.3%; 95% CI 52.9–57.7%)). Data from the BRFSS showed that endoscopic screening rates increased among individuals with a low household income (USD 10,000–USD 15,000). Data from the MEPS showed that among individuals aged 65–75 (*n* = 958), a statistically significant change in the prevalence of CRC screening post-ACA (2011–2012) was noted among those whose income was <125% of the FPL compared to pre-ACA screening prevalence (2009–2010; 5.7% point change; 95% CI 0.18–11.3). Although this increase may not be completely attributable to the ACA provisions on CRC screening, the increased coverage of the socioeconomically disadvantaged and the removal of cost-sharing are direct consequences of the ACA. Of note, although the implementation of the ACA should have led to the delivery of preventive health services to the public without charge, cost-sharing for colonoscopies after non-invasive screening tests still occurred in 48.2% of patients with commercial insurance and 77.9% of patients with Medicare coverage based on the analysis of the MarketScan Commercial database and the Medicare Supplemental administrative claims databases from 1 January 2014 to 31 July 2019 [32].

Disparities in colorectal cancer (CRC) screening rates between commercially insured individuals and those covered by Medicare or Medicaid likely reflect differences in financial and structural barriers. In Medicaid populations, lower provider reimbursement has been shown to limit provider participation and access to care [33]. Medicare beneficiaries without supplemental insurance may still face cost-sharing for procedures that convert from screening to diagnostic, which can deter participation [34]. Additionally, while insurance coverage is necessary, it is not sufficient to ensure screening—uptake remains influenced by system-level factors such as care coordination, patient navigation, and proactive outreach.

## 5. Equity in CRC Screening

Although consistent signals suggest that CRC screening rates have increased, the question of the equitable delivery of CRC screening remains unanswered. Data from the BRFSS shows that CRC screening rates increased disproportionately after the implementation of the ACA. In 2019, only 67.1% of adults aged 50–75 had undergone screening in the US, a 14% increase since 2008 [35]. When stratified by race, none of the communities had reached the 80% screening put forth by the National Colorectal Cancer Roundtable (NCCRT) [36]; the lowest screening rates were among Hispanics, compared to the highest among Whites; this translated into a 17% disparity rate. This study also showed that colonoscopy was the most common screening modality each year from 2008 to 2016. An evaluation of the data from 2018 showed that 54% of Medicaid beneficiaries were up-to-date with CRC screening, compared to 73% of adults with Medicare and 80% of adults with Medicare and commercial insurance combined [37]. As mentioned above, Medicaid programs were not required to report CRC screening rates as a performance metric during the timeframe of these studies. More recent data (2021) from the NHIS suggests that 59% of individuals aged 45 years or older had received a CRC screening [38]; low screening prevalence was noted among individuals of 45–49 years of age (20%), those with residence in the US for <10 years (29%), those with a lack of insurance (21%), and individuals with less than a high school-level education (49%).

Racial inequities in CRC screening are prevalent with limited data on effective mitigation of such disparities. Black individuals have the highest CRC incidence and mortality of all racial groups in the US (7.4 more CRC diagnoses for men; 4.5 more for women per 100,000; 5.3 more deaths for men; 2.9 more for women per 100,000 compared to their White counterparts) [39]. Simulated data have suggested that a major proportion of Black–White disparity in CRC screening (42%) and CRC incidence (19%) are attributable to screening differences [40]. However, data from the BRFSS from 2018 suggests that the historical gap in screening among Blacks and Whites has been narrowed [41]; 70% and 71% of Blacks and Whites had received a CRC screening in 2018 at a national level. American Indian/Alaskan Native (AI/AN) individuals continue to have higher CRC incidence, particularly among women (4.5 additional CRC diagnoses per 100,000 than their White counterparts) [39,42]. CRC-related mortality is also higher for both AI/AN men (0.7 more deaths per 100,000) and women (1.5 more deaths per 100,000) than their White counterparts. Interestingly, Hispanic men (3 fewer diagnoses per 100,000) and women (3.7 fewer diagnoses per 100,000) have lower CRC diagnoses and mortality (2.1 fewer for men; 2.6 fewer for women per 100,000) than their White counterparts [39]. Hispanics are nonetheless more likely to be diagnosed with advanced-stage CRC; it is unclear whether this disparity is targetable from a screening perspective and should be a matter of further study.

## 6. The ACA and CRC Screening—Overall Policy Evaluation

### 6.1. Economic Criteria

The ACA increased access to preventive health services and increases in CRC screening prevalence have been noted, which are most prominent among individuals of low socioeconomic status (e.g., individuals who benefited from Medicaid and the Appalachian population [22]).

### 6.2. Equity Criteria

Despite a notable increase in CRC screening prevalence among the socioeconomically disadvantaged, a disproportionate increase in prevalence has been demonstrated when stratified by race, with the highest rates in Whites and the lowest in Hispanics [35].

### 6.3. Technical Criteria

Although CRC screening has increased overall, it did not reach the 80% target put forth by the NCCRT [36]. Colonoscopies were the most utilized screening modality, and suboptimal utilization of stool-based screening tests may partly explain this disparity and may even pave the way for future policymaking as these tests are easy to administer (i.e., at home and mailed in) and do not impose a high burden on the patient.

### 6.4. Political Criteria

The ACA provisions on CRC screenings are in line with the values of population well-being since screening prevents morbidity and mortality associated with one of the most common cancers in the US. These provisions were supported by policymakers, healthcare personnel, and medical associations and colleges such as the American College of Gastroenterology, the American Association of Gastroenterology, the American College of Physicians, and the American Cancer Society.

### 6.5. Administrative Criteria

The infrastructure for CRC screening was available at the time of ACA implementation. With the ACA provisions, the infrastructure was further solidified as it financed the operation of a screening procedure, which also generated resources for administrative and support staff.

## 7. Policy Interventions for Colorectal Cancer Screening After the ACA

In 2020, an advisory by the Federal Government emerged on phasing out coinsurance between 2022 and 2030 under the “Removing Barriers to Colorectal Cancer Screening Act of 2020” [43]. On 1 January 2022, Section 1833(a) of the “Consolidated Appropriations Act” was amended to gradually reduce coinsurance of procedures performed in connection to CRC screening tests for Medicare beneficiaries starting in January 2023, with the goal of no coinsurance incurred by individuals by 2030 [44]. On 10 January 2022, the Federal Government provided updated guidance that requires commercial insurers to remove patient cost-sharing for colonoscopies classified as diagnostic after a positive CRC screening test, beginning 31 May 2022 [45]. These policy-level interventions may lead to improved coverage and delivery of diagnostic colonoscopies while eliminating surprise billing practices for colonoscopies. Furthermore, in 2021, the Centers for Medicare and Medicaid Services (CMS) Quality Measures Voting Members recommended that CRC screening be added to the CMS Medicaid Adult Core Set of Quality Measures [37]; this would require Medicaid providers to report CRC screening as a quality metric and could have a positive impact on CRC screening rates among Medicaid beneficiaries.

## 8. Acceptability of CRC Screening Modalities

Data on patient preference seems to consistently weigh in favor of non-invasive screening tests over a colonoscopy. A survey of 1595 individuals who were provided a choice between CRC screening modalities showed a preference for multitarget stool-DNA testing (mt-sDNA), fecal immunochemical testing (FIT), and fecal occult blood testing (FOBT) over a colonoscopy [46]. These findings were corroborated in a study of 1000 participants, 456 of whom were aged 40–49, providing insight into the preferences of younger individuals now eligible for CRC screening [47]. Stool-based CRC screening has also been prevalent in resource-limited settings, as is evident by data from the CHC in Medicaid expansion states [20]. FIT-based screening has now been shown to be non-inferior to colonoscopy screening in a first randomized trial comparing the two modalities [48]; participation in CRC screening was higher among individuals invited for FIT screening.

In contrast, a colonoscopy was the preferred screening modality for gastroenterologists and Primary Care Physicians, albeit with some variation and preference for stool-based tests by a small yet notable proportion of Primary Care Physicians (PCPs) [49]. This patient-provider discrepancy may be a barrier to increasing the CRC screening rate. It is difficult to reconcile these findings and understand the patient–physician discrepancy; one possible explanation is the difference in health literacy, where gastroenterologists recognize the diagnostic and therapeutic role of a colonoscopy, which also provides a relatively longer screening-free interval to patients. In contrast, PCPs may favor stool-based testing as this may be a trackable quality metric of their clinical performance, and patients likely prefer the convenience of stool-based testing. Another possible explanation is the financial incentive of gastroenterologists to promote a colonoscopy over stool-based tests, either consciously or subconsciously. The ACA does not favor one CRC screening strategy over another but lends support to promoting CRC screening, be it by utilizing colonoscopies or by promoting a two-step screening strategy.

## 9. The Benefits and Shortcomings of a Two-Step Approach to CRC Screening

At this juncture, it is worthwhile to briefly describe the following:

1. Screening colonoscopy: A colonoscopy may be classified as a screening test if it is the index test being performed for CRC screening and is not preceded by a positive non-invasive test.
2. Diagnostic colonoscopy: A colonoscopy is classified as diagnostic for a variety of reasons. For this discussion, a colonoscopy is considered diagnostic rather than a screening test when it is preceded by a positive non-invasive test such as a positive stool test. Efforts are underway to label follow-up colonoscopies as screening rather than diagnostic after positive non-endoscopic CRC screening tests.
3. Therapeutic colonoscopy: A colonoscopy is considered therapeutic when a polyp resection or intervention of a similar nature is performed. A screening or diagnostic colonoscopy may convert to a therapeutic colonoscopy when a polyp is removed.

It is also worthwhile to put into perspective the efforts that go into the completion of a colonoscopy:

1. Health system-related factors: The delivery of instructions on diet and bowel preparation, adjusted by individual health literacy, acknowledging reservations and stigma that may exist surrounding a procedure performed trans-anally. Also worth considering is the administrative burden of scheduling and triaging colonoscopies; in a resource-limited health system, this variable is critical to the timely completion of a colonoscopy.
2. Patient-related factors: Compliance with instructions provided, including adherence to a clear liquid diet and the completion of bowel preparation, social support for logistical factors, taking a day off work and household responsibilities, and preference for endoscopists’ gender [9,10].
3. Comorbidity-related factors: Obtaining peri-operative clearances; this may include appointments with consultants, the completion of tests required to give clearance, holding or bridging anti-coagulation therapy, and the follow-up appointment(s) for final pre-operative clearance.
4. Logistical factors: Arranging transport in cases where sedation is used, a common practice for colonoscopies performed within the US. This may also entail arranging and paying for a child-care or an adult-care provider for the family member(s).

As mentioned above, despite it being a labor-intensive modality that may not be required in all cases (but remains the gold standard option), colonoscopies were the most utilized screening modality over the past three decades in the US (Figure 1). FOBT, FIT, and mt-sDNA are supported by a sizeable body of evidence on their screening utility and patient preference and allow the capturing of individuals who would benefit the most from a colonoscopy. Colonoscopy after a positive stool-based CRC screening test is likely to detect more adenomatous polyps, advanced adenomatous polyps, and CRC [50] and may carry a higher survival benefit, with more life-years gained and CRCs averted compared to an average-risk screening colonoscopy [51]. The adenoma detection rate is also higher among patients with a positive FIT test (59.3%) and a positive FOBT test (53.8%) compared to average-risk individuals [4]. The COVID-19 pandemic imposed a new set of challenges that evolved the arena of CRC screening in favor of non-invasive testing; 2018–2020 data from BRFSS showed a 7% increase in stool-based CRC screening (adjusted prevalence ratio (aPR) 1.07; 95% CI 1.02, 1.12) [52]; this increase was offset by a 16% decrease in screening colonoscopies (aPR 0.84; 95% CI 0.82–0.88), and the overall CRC screening prevalence remained steady. A similar trend was seen in an analysis of 2019–2021 data from the NHIS [53]; CRC screening prevalence was unchanged, with an increase in stool-based testing from 7% to 10.3% (aPR 1.44; 95% CI 1.31, 1.58), which was offset by a decrease in colonoscopy rates from 15.5% to 13.8% (aPR 0.88; 95% CI 0.83, 0.95). Adherence to screening has socioeconomic underpinnings; data suggests that individuals with commercial insurance, the elderly (who have Medicare coverage), patients from higher income groups, and patients of gastroenterologists (i.e., those who have access and the means to benefit from specialty care) had a ≥70% adherence rate to mt-sDNA testing, whereas the adherence rate for Medicaid beneficiaries was 52% [54]. Such findings suggest that increasing national CRC rates necessitate the utilization of a socioeconomic lens for durable results.

A two-step screening approach, though seemingly less labor intensive for most patients, presents its own challenges, including the completion of a follow-up diagnostic colonoscopy. It is prudent to highlight that, under a two-step strategy, CRC screening is rendered incomplete without a follow-up colonoscopy. Delays in the timely completion of a follow-up colonoscopy have been shown to increase the risk of diagnosing CRC and advanced CRC [50]. Non-compliance with a colonoscopy after a positive FIT has been shown to double the risk of dying from CRC [55].

Studies have consistently reported suboptimal adherence and completion of a follow-up colonoscopy after a positive stool-based CRC screening test. Data from eight Federally Qualified Health Centers from 1229 individuals with an abnormal FIT test showed that 89% of the patients had a follow-up colonoscopy referral, and only 44% had colonoscopy completion [56]. A study of the data from 12 Veteran’s Affairs sites showed that merely 62.1% of patients with positive FIT tests completed a follow-up colonoscopy; of the 37.9% of patients who did not undergo a follow-up colonoscopy, most (35.2%) declined a colonoscopy, whereas 12% had scheduling issues and 2.9% had transportation issues [57]. A study of the Population-Based Research Optimizing Screening through Personalized Regimens (PROSPR) cohort members showed that of the 1267 individuals with a positive FIT test, only 42.3% were able to undergo a follow-up colonoscopy [58]. Although the exact causes of not having a follow-up colonoscopy were not provided, a majority of the patients did not show up for their pre-procedural evaluation appointments, hinting at either a lapse of communication at a system level or potential logistical issues at an individual level. Another study of the North Texas safety-net health system showed that 45% of the patients with a positive FIT test did not complete a follow-up colonoscopy; 38% of these patients attributed this to insurance-related health challenges and 29% to social barriers such as transportation [59]. Breen et al. studied the time to a follow-up colonoscopy for 35,000 patients with a positive FIT test; they showed that patients with Medicaid were less likely than those with commercial insurance to complete a follow-up colonoscopy. Medicaid patients also had a longer average time to follow-up [60]. As mentioned above, cost-sharing practices continued years after the enactment of the ACA and were likely to have been an additional barrier in the way of a two-step screening strategy [32].

These data suggest that cost-sharing issues, logistical issues, and patients declining a colonoscopy are among common, identifiable factors that lead to low rates of the completion of a follow-up colonoscopy after a positive stool-based test. Modeling the study of CRC screening suggests that in low screening adherence environments, a 45% first-step adherence and 80% adherence to a follow-up colonoscopy among patients with a positive FIT is likely cost-effective [61].

## 10. CRC Screening and Survival

Data from the Surveillance, Epidemiology, and End Results Program (SEER) cancer registry showed an estimated 8% increase in the diagnosis of early-stage CRC per year among American seniors from 2011 to 2013 (an estimated additional 8400 early-stage cancer diagnoses) [62]. Data from the National Cancer Database between pre-ACA (2011–2013) and post-ACA (second and fourth quarter of 2014) terms showed a small but statistically significant increase in early-stage CRC diagnosis among Medicaid expansion states (relative percent change: 4.1; absolute percent change: 0.8%; 0–1.6%) whereas a nonsignificant increase in early-stage CRC diagnoses was observed in states without Medicaid expansion [63]. Of note, the National Cancer Database categorizes stage I CRC as an early-stage disease, whereas the SEER categorizes stage I and II CRC as an early-stage disease.

Data from more than 2 million patients aged ≥50 years from the Kaiser Permanente Northern California database between 2003 and 2016 (pre-ACA: January 2003–March 2010; post-ACA: April 2010–December 2016) showed that around the time the ACA was enacted, there was a 17% (95% CI 10–23%) decrease in incidence of CRC, followed by a 3% (95% CI 0–7%) annual decrease thereafter [64]. This translated into an absolute post-ACA incidence rate ratio (IRR) of 0.96 (95% CI 0.95–0.98). Furthermore, a 15% (95% CI 4–25%) decrease in CRC-related mortality was also noted, followed by a 6% (95% CI 1–12%) annual decrease thereafter (IRR 0.96; 95% CI 0.94–0.99). It is worth noting that the efforts to increase CRC screening were driven as a response to the implementation of CRC screening as a Healthcare Effectiveness Data and Information Set (HEDIS) metric, coupled with a large-scale mailed FIT testing initiative.

In 2022, the results of a pragmatic, randomized trial on the utility of a colonoscopy as a screening tool among individuals aged 55–64 from Scandinavian countries were published [65]. Of the 84,585 individuals in the trial, 28,220 were randomized to receive an invitation for a screening colonoscopy; 11,843 of these patients (42%) underwent a screening colonoscopy. The risk of CRC was lower among those invited to undergo a colonoscopy over a 10-year follow-up (risk ratio: 0.82; 95% CI 0.70–0.93) in the intention to treat analysis. However, no difference in death from CRC was noted in the ITT analysis. In the per-protocol analysis, however, the risk of death from CRC was lower (0.15%) in the screening arm compared to the control group (0.30; risk ratio: 0.50; 95% CI 0.27–0.77). These findings suggest that the mortality benefit from a screening intervention is dependent on the successful completion of the intervention, which in this case was a screening colonoscopy.

Interestingly, despite higher utilization of a screening colonoscopy in the US, the age-adjusted mortality rate from CRC in the US is among the highest in the developed world, which is likely a projection of the lack of screening in a major proportion of Americans as outlined by the NCCRT and highlighted above. This further underscores that the population-level impact on CRC mortality necessitates the large-scale administration and completion of screening; in this context, the best screening modality is one that is acceptable by the masses and can be completed in a cost-effective manner on a societal scale.

## 11. CRC Screening and Health Policy—Bottom Line

CRC screening rates have undoubtedly increased over the past decades and were on an increasing trend in the pre-ACA era; from 2000 to 2019, a linear increase in CRC screening prevalence among individuals aged 50–75 can be seen from CDC data. With the enactment of the ACA, the socioeconomically disadvantaged may have gained the most benefit by gaining access to preventive healthcare delivery through Medicaid. However, the long-term increase in CRC screening through Medicaid is not obvious. In light of such data, it is reasonable to question if the ACA had as prominent of an impact on CRC screening as we would like to believe [40]. One could argue that watchful waiting and assessment of the impact of policies such as the ACA on CRC screening and mortality may be appropriate to better capture time-dependent events. Alternatively, in light of such data, a reengineering of our approach to CRC screening may also be reasonable.

In the US, colonoscopies have been utilized relatively more than in other industrialized nations for CRC screening, whereas the use of stool-based tests has varied and arguably has been suboptimal. A limited body of evidence suggests that stool-based testing is the favored screening modality for patients since it carries the advantage of administration within the comfort of one’s home, a characteristic that may be appealing to the growing number of younger individuals (45–49 years of age) who are now eligible for screening. Unfortunately, ~50% of patients with positive stool-based CRC screening tests do not complete the requisite follow-up colonoscopy [29,30,31,32], deeming them unscreened with double the risk of dying from CRC [55]. While the aforementioned is multifactorial, cost-sharing practices, particularly for follow-up colonoscopies after an abnormal non-invasive test, may have been partly to blame. Policies to eliminate cost-sharing have been reiterated in recent years; the implementation of such policies may render a two-step strategy to be cost-effective in modestly increasing CRC screening if the completion of a follow-up colonoscopy is assured [66]. There are unfortunately no policies in place to ensure the timely completion of a follow-up diagnostic colonoscopy nor quality metrics monitoring the process to the completion of such a colonoscopy; this also hinders the promotion of a two-step screening strategy.

## 12. Conclusions and Policy Recommendations

**Figure 2:**
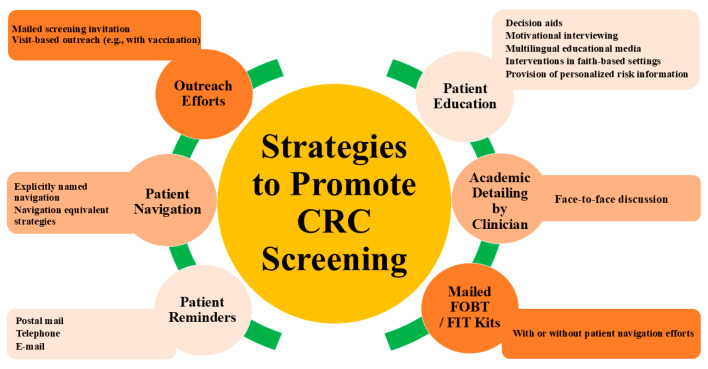
Summary of evidence-based strategies to improve CRC screening uptake.

**Figure 3:**
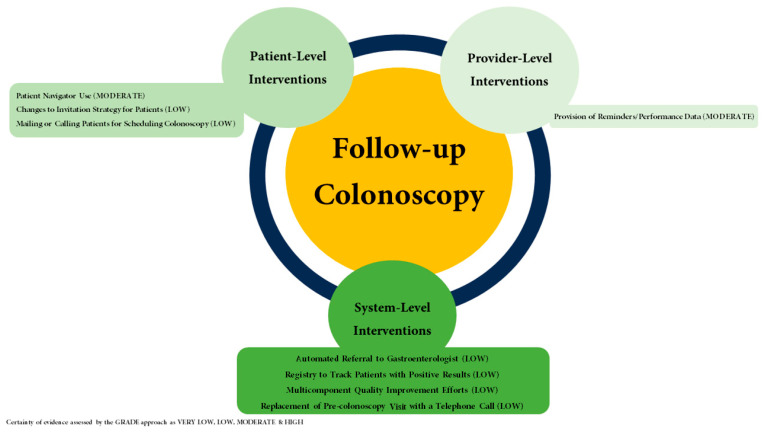
Evidence-based strategies to improve follow-up colonoscopy completion rates.

To reach the target of an 80% screening rate, multilevel policies are needed beyond the provisions of the ACA. Such endeavors should involve community members and stakeholders, address barriers to screening delivery, be sustainable over time, and be replicable for a variety of healthcare settings. Our recommendations, in order of priority, are as follows:

1. Establishing the proportion of patients with an abnormal non-invasive screening test with a completed follow-up colonoscopy as a HEDIS quality metric. The US Multisociety Task Force on Colorectal Cancer (USMSTF) recommends at least an 80% completion rate of colonoscopy for patients with a positive FIT [2].
2. Tracking time to a follow-up colonoscopy after a positive index screening test and establishing time to a follow-up colonoscopy as a HEDIS quality metric, with the aim to complete a follow-up colonoscopy within 9 months [50].
3. The removal of all cost-sharing for a follow-up colonoscopy. This includes addressing insurance-related issues as has been performed under the “Removing Barriers to Colorectal Cancer Screening Act of 2020”. The implementation of such guidance does not extend to Medicare yet and should be a topic of future policymaking debates.
4. Enabling reimbursement for outreach efforts with established efficacy in the completion of index CRC screening, the completion of a follow-up colonoscopy after a non-invasive screening test, and the completion of follow-up screening after a negative index screening test (Figure 2 and Figure 3) [67,68].

These policy recommendations address factors that can be implemented at a national level (e.g., removal of cost-sharing and improving reimbursement practices) and those that will benefit from adoption and support by national cancer and gastroenterology societies (e.g., providing support for the establishment of HEDIS quality metrics), as well as those that can be implemented at a health system level (e.g., tracking of quality metrics).

The constitutional legitimacy of providing healthcare, particularly preventive health services to the nation, should be upheld with rigor and promoted as a human right that can withstand any and all challenges that may come its way. As healthcare providers, we serve to provide guidance to our patients. CRC screening is one such task where patient responsibility, and the interplay of physician–patient relationships, plays a vital role. As such, we must leverage the value of our relationships to tailor the message of screening practices to patients’ values and preferences.

## Figures and Tables

**Table 1:** United States Preventive Services Task Force recommendations for colorectal cancer screening.

Age Range	Recommendation	Grade	Screening Options
45–49 years	Colorectal cancer screening is recommended for average-risk individuals	B	Colonoscopy—every 10 years High-sensitivity gFOBT—every year FIT—every year sDNA-FIT—every 1–3 years CT colonography—every 5 years Flexible sigmoidoscopy—every 5 years
50–75 years	Colorectal cancer screening is recommended for average-risk individuals	A	
76–85 years	Selectively offer colorectal cancer screening	C	

## Data Availability

The data used for this manuscript is publicly available.
